# Seasonal Variations of Pollinator Assemblages among Urban and Rural Habitats: A Comparative Approach Using a Standardized Plant Community

**DOI:** 10.3390/insects12030199

**Published:** 2021-02-27

**Authors:** Vincent Zaninotto, Adrien Perrard, Olivier Babiar, Amandine Hansart, Cécile Hignard, Isabelle Dajoz

**Affiliations:** 1 Institute of Ecology and Environmental Sciences-Paris (iEES-Paris), Sorbonne Université, CNRS, IRD, INRAE, Université de Paris, UPEC, 4 Place Jussieu, 75005 Paris, France; adrien.perrard@univ-paris-diderot.fr (A.P.); isabelle.dajoz@univ-paris-diderot.fr (I.D.); 2 Direction des Espaces verts et de l’Environnement, Mairie de Paris, 103 Avenue de France, 75013 Paris, France; 3 Station d’Écologie Forestière, Université de Paris, Route de la tour Dénécourt, 77300 Fontainebleau, France; olivier.babiar@univ-paris-diderot.fr (O.B.); cecile.hignard@univ-paris-diderot.fr (C.H.); 4 Centre de Recherche en Écologie Expérimentale et Prédictive (CEREEP-Ecotron IleDeFrance), Département de biologie, École Normale Supérieure, PSL University, CNRS, UMS 3194, 11 Chemin de Busseau, 77140 Saint-Pierre-lès-Nemours, France; amandine.hansart@ens.fr

**Keywords:** pollinator communities, functional traits, urban ecology, body size, inter-tegular distance, seasonality, *Sinapis alba*, *Lotus corniculatus*

## Abstract

**Simple Summary:**

Urbanization modifies the composition of all biological communities, including insect pollinator communities, but what is filtered out? To answer this question, we compared the pollinators and their morphological and behavioral characteristics between Paris green spaces and nearby rural grasslands. We monitored the pollinators foraging on identical plant plots in these two environments for two years, and from spring to fall. Pollinators in the city were relatively less diverse than their rural counterparts. They comprised fewer bees belonging to solitary or ground-nesting species, but the bees had a larger body size overall. These data add to the body of evidence of a filtering of pollinator communities by the urban environment, partly because the abundance and distribution of nesting and feeding resources are modified. Since the diversity of pollinators is important for plant pollination, such effects must be considered in order to preserve the insect pollinator community and maintain the pollination function despite the increasing urbanization of our landscapes.

**Abstract:**

Even though urban green spaces may host a relatively high diversity of wild bees, urban environments impact the pollinator taxonomic and functional diversity in a way that is still misunderstood. Here, we provide an assessment of the taxonomic and functional composition of pollinator assemblages and their response to urbanization in the Paris region (France). We performed a spring-to-fall survey of insect pollinators in green spaces embedded in a dense urban matrix and in rural grasslands, using a plant setup standardized across sites and throughout the seasons. We compared pollinator species composition and the occurrence of bee functional traits over the two habitats. There was no difference in species richness between habitats, though urban assemblages were dominated by very abundant generalist species and displayed a lower evenness. They also included fewer brood parasitic, solitary or ground-nesting bees. Overall, bees tended to be larger in the city than in the semi-natural grasslands, and this trait exhibited seasonal variations. The urban environment filters out some life history traits of insect pollinators and alters their seasonal patterns, likely as a result of the fragmentation and scarcity of feeding and nesting resources. This could have repercussions on pollination networks and the efficiency of the pollination function.

## 1. Introduction

Urbanization changes the environment in many ways that affect organisms [1], although some species are better equipped than others [2]. We know that pollinators are affected [3], but it is not clear how these communities respond to urban constraints. To answer this question, we analyzed the pollinators monitored in an experimental plot of flowering plants over two years in rural and urban habitats.

Urbanization operates an environmental filtering on species assemblages in general [1], including insect pollinator communities [4]. However, there is no consensus about the effect of the urban habitat on pollinator species diversity. For example, in Great Britain, both the diversity and abundance of pollinators are negatively associated with urbanization [5], though urban habitats may support more diverse wild bee communities than agricultural landscapes [6]. Meanwhile, pollinator assemblages in downtown Paris (France) appear less diverse than those recorded in the surrounding landscapes, whether semi-natural or agricultural [7]. Indeed, densely urbanized landscapes do not seem to be suitable to host diverse communities of insect pollinators as their nesting and feeding habitats tend to become scarce and fragmented [8,9].

In addition to examining species diversity, it is worth assessing how the functional diversity of pollinator assemblages responds to urbanization. Functional approaches help to understand the link between biodiversity and habitat constraints [10]. As a matter of fact, urban habitats also seem to apply a filtering on functional traits of insect pollinators. There is widespread evidence that urban pollinator communities are less functionally diverse than their rural counterparts, as these communities mainly host generalist flower visitors [6,11,12]. In France in particular, urbanization is associated with a shift in community composition, suggesting a large-scale functional homogenization of flower visitor assemblages [13]. Indeed, traits such as nesting habits and lectism seem to be strongly affected by urbanization. Urban habitats would benefit cavity-nesting bees and polylectic species, while they appear unfit for ground-nesting bees and oligolectic insect pollinator species [14,15,16,17,18].

Among other functional traits, body size is of particular importance, since it is related to environmental characteristics that are strongly influenced by urbanization, such as floral resource quantity, habitat fragmentation and climatic conditions. On the one hand, size is related to resource availability, as undernutrition at the larval stage can result in smaller adults. In addition, small-bodied insects need less resources to survive and reproduce, so they may be advantaged in resource-poor urban environments [10,19]. On the other hand, body size is associated with foraging range: large-bodied insects fly longer distances [20]. In the city, the fragmented habitat could advantage large-bodied bees that can easily fly from one patch to another [21,22]; however, resources are often pooled in green spaces which could render long-distance foraging unnecessary [10]. There is evidence for reduced body size of bees in urban habitats, both at the species level [23] and community level [14,15,24], though it is disputed [22]. In addition, body size could also be influenced by climatic conditions, as it may be involved in thermic resistance and regulation, although there is no clear general temperature–size relationship in ectotherms [25,26,27]. Hence, Gerard et al. [28] reported latitudinal clines in the size of wild bees following Bergman’s rule (size increases with latitude) across Europe, although they found the opposite climate–size relation for some genera such as *Bombus*. Last, Osorio-Canadas et al. [29] revealed a seasonal pattern in the body size of wild bees at the community level, with small sizes becoming more prevalent during summer. To our knowledge, how bee body size varies throughout the seasons in urban habitats remains to be tested. Indeed, urban habitat characteristics such as the year-long availability of ornamental floral resources [30,31] and their fragmented spatial distribution should buffer variations in body size throughout the seasons. On the contrary, the warmer conditions due to the urban heat island [32] could more strongly constrain the size of individuals by requiring increased thermal resistance and regulation capacities, especially during the hottest months.

Here, we compare the species and functional diversities of rural and urban communities of pollinating insects, taking advantage of an experimental design where a standardized plant assemblage was set up in several rural and urban locations within the same biogeographical region (Ile-de-France region, France). We focus on the following questions: (i) What are the differences in the species assemblage and diversity visiting the experimental plants between rural and urban pollinator communities? (ii) Focusing on wild bee communities visiting the experimental plants, how do the functional traits of these urban and rural communities vary throughout the year?

We expect differences in overall species assemblage composition between the urban and rural communities visiting both plant species. For instance, we expect eusocial generalist pollinators such as bumble bees and managed honey bees to represent a larger proportion of the urban community [33], while syrphid flies should be more abundant and diverse in rural areas [34,35]. Further, on both plant species, greater species richness and diversity of pollinators are expected in the rural habitat in comparison with the urban one—here, downtown Paris, one of the most densely populated cities in the world [36]. Concerning the functional diversity of wild bee assemblages, we also expect differences in bee levels of sociality, lectism, nesting habits and proportion of brood parasites. The urban habitat is likely to host more eusocial species but fewer ground-nesting, brood parasitic and oligolectic species than the rural habitat, and these differences should be recorded throughout the season. Finally, we expect differences in bee body size between the two habitats, estimated through the inter-tegular distance (ITD). In addition, seasonal body size variations at the community level may differ between the two habitats. All of these potential differences between urban and rural pollinator communities are likely to be reflected in the assemblage of flower visitors of our plant setups.

## 2. Materials and Methods

Sampling was conducted for two consecutive years in six (March to July 2017) and four (March to November 2018) locations in a dense urban habitat and semi-natural habitats (hereafter referred to as “rural”), all within the Ile-de-France region (France). Urban sites were located in downtown Paris (20,754.5 hab./km² [37]). They were set up in green spaces with lawns and ornamental flowerbeds that were not treated with pesticides, and surrounded by a dense urban matrix [38]. Meanwhile, rural sites were all located in the Seine-et-Marne administrative department (50–64 km from Paris) near the towns of Fontainebleau (86.5 hab./km²) and Saint-Pierre-lès-Nemours (253.6 hab./km²) [37]. They were set up in semi-natural grasslands, free of pesticides and surrounded by forests [38]. The same sites were used throughout both years, with the exception of two sites that were not operational in 2018 (one in each habitat type). Details on experimental site surroundings can be found in Table A1.

Our pollinator surveys were carried out on replicates of a plant setup that was standardized across sites and over the course of the seasons. This enabled us to capture flower visitors over time without any variation in the overall attractiveness of the plants and their floral traits, even though the collected insects represent only a subset of the whole pollinator community. In addition, this sampling method did not introduce any size bias, unlike with pan traps [39]. In each experimental site, two 1.6 × 1.2 m plots were set up side by side in a grassland area, each containing one of the two focal plant species (the Brassicaceae *Sinapis alba* L. and the Fabaceae *Lotus corniculatus* L.). *Sinapis alba* L. is an annual forb growing along roads, in wastelands or near crops and is considered naturalized in the Ile-de-France region [40]. *Lotus corniculatus* L. is a perennial plant widespread in grasslands and disturbed habitats [41] and is native to the Ile-de-France region [42,43]. Both species bear yellow flowers with contrasting floral morphologies: *S. alba* has flat corollas with floral rewards accessible to pollinators with short mouthparts [41,44], whereas *L. corniculatus* has deep corollas with floral rewards mainly accessible to pollinators with long mouthparts [45]. This allowed us to attract a large range of insect pollinators with diverse mouthpart morphologies and floral resource requirements [44]. Prior to their installation in the field, plants were grown in individual pots under insect-proof greenhouse conditions (temperature: 20 °C; photoperiod: 16 h of day; 12 cm diameter plastic pots filled with peat-enriched sowing soil: 180 g.m-3 N, 450 g.m-3 P2O5, 90 g.m-3 K2O) and were installed in the experimental plots when they began flowering. In each plot, 20 pots containing one plant of the same species were buried in four rows of five, each plant being spaced from others by 25 cm in all directions. The floral cover was kept constant throughout the study period (March to November) by regular watering and by renewing the plants every twenty days, replacing them with fresh plants from the greenhouse.

Pollinator surveys in 2017 were limited to spring and early summer (March–July). In 2018, sampling was extended to summer and fall (March–November), with an interruption between mid-July and mid-August due to strong heat waves that were detrimental to the plant sets. Twice a week, and within each locality, five-minute insect pollinator sampling sessions were carried out on each of the two plant species. All sites were sampled on the same days between 11:00 a.m. and 3:00 p.m., in alternating order. A total of 33 five-minute sessions per site were conducted in 2017, evenly distributed over 17 weeks; and 56 five-minute sessions per site in 2018, evenly distributed over 31 weeks. During these sessions, all insects coming into contact with the fertile parts of the plants were captured, using plastic boxes. The insects were then euthanized by transferring them to vials saturated with ethyl acetate vapors before being processed in the laboratory, where each specimen was labeled, pinned and identified down to the genus, then sent to specialists for species identification. All specimens are now kept in the collection of the iEES laboratory, 4 place Jussieu, 75005 Paris, France.

**Figure 1:**
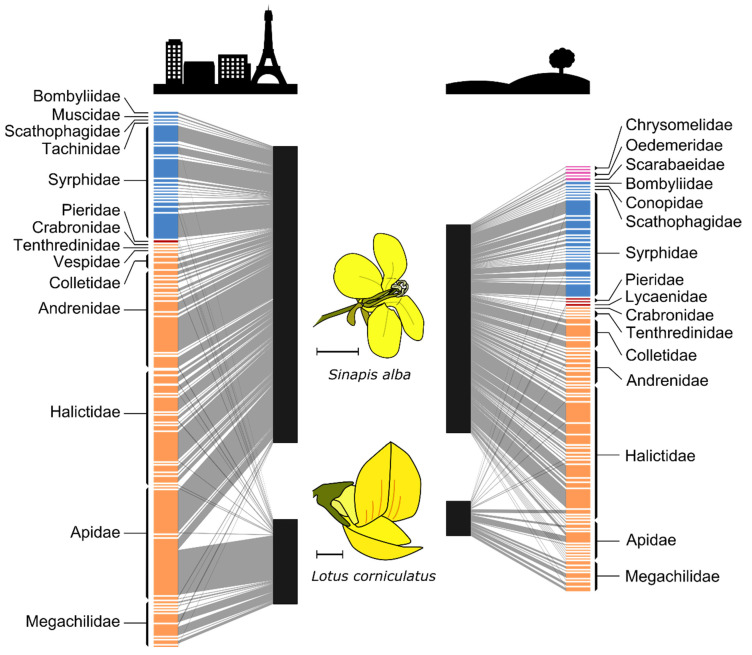
Plant–pollinator interaction networks in both habitats (Urban: left; Rural: right), centered on our two plant species (*Sinapis alba*: top; *Lotus corniculatus*: bottom). Links represent the number of interactions between flower visitor species (colored boxes, labeled at the family level) and each plant species (black boxes). Colors represent insect orders: purple = Coleoptera; blue = Diptera; red = Lepidoptera; orange = Hymenoptera. The scale bar of the flower diagrams measures 5 mm.

**Figure A1:**
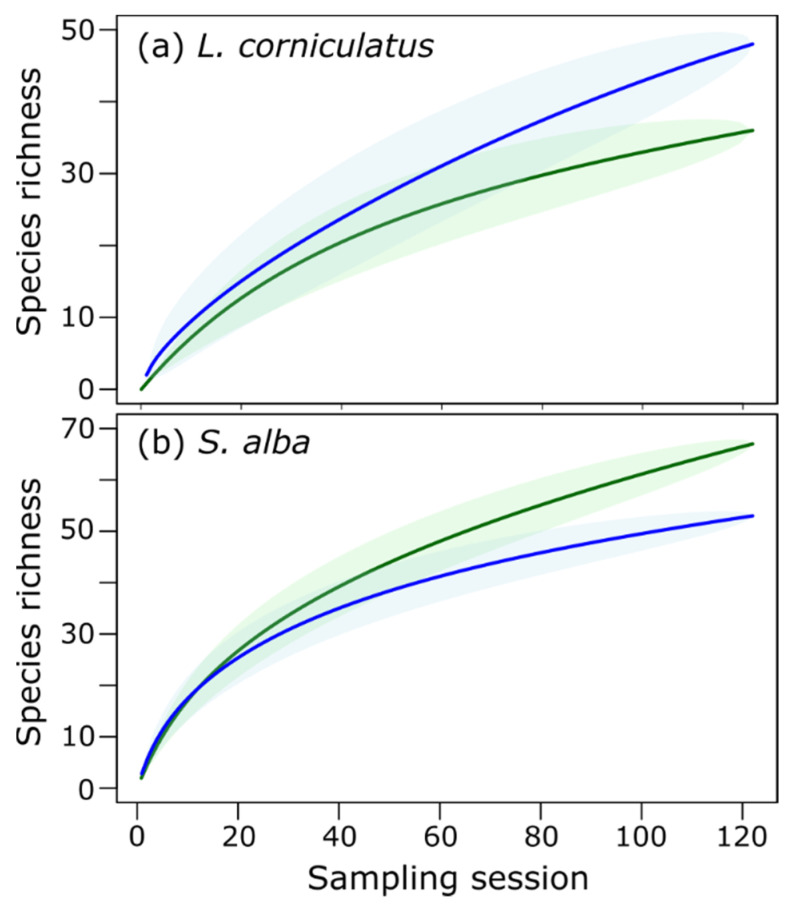
Rarefaction curves of insect pollinator species on both plant species, in both habitats (Urban: blue; Rural: green).

All data analyses were carried out using the R software version 4.0.3 [46]. First, interaction networks (Figure 1) and rarefaction curves (Figure A1) were plotted using, respectively, the “plotweb” function of the “bipartite” package [47], and the “specaccum” function of the “vegan” package [48]. Proportions of individuals belonging to the main families of pollinators (number of individuals > 5) were compared between the two habitats using a chi-squared contingency table test (“chisq.test” function).

Species richness and Simpson’s diversity index of flower visitors were calculated for each plant species, locality and year with the “diversity” function of the “vegan” package [48]. Values taken by Simpson’s diversity index vary between 0 and 1, with low values recorded for communities dominated by a few, very abundant species. In contrast, functional diversity was estimated only on wild bees, using Rao’s quadratic entropy (“dbFD” function of the “FD” package [49]) calculated over five traits: lectism, sociality level, nesting habits, brood parasitism status and species average inter-tegular distance (mm). Rao’s quadratic entropy provides a joint assessment of functional richness and functional dispersion, with low values indicating low functional diversity [50]. To test whether values taken by these diversity indices per plant species, locality and year (n = 20) differed between the two habitats, we used beta-regression generalized linear mixed models (hereafter glmms) (R package “glmmTMB” [51]). We defined the plant species (“*Lotus corniculatus*” vs. “*Sinapis alba*”), the habitat (“Urban” vs. “Rural”) and their interaction, as well as the year (2017 or 2018), as fixed effects, and the sampling site as a random effect. Species richness was modeled with a Poisson error distribution, whereas Simpson’s index and Rao’s quadratic entropy were modeled with a Gaussian error distribution. In order to complement our data with qualitative information on species rarity, we also compared the amount of specimens from species listed as endangered or notable in the IUCN red list [52] and local ZNIEFF lists [53,54].

Traits data of wild bees (lectism, brood parasitism status, sociality level and nesting habits) were recovered from Gerard et al. [28]. We used glmms to characterize the seasonal patterns in the proportions of specimens belonging to eusocial species (both primitively eusocial Halictidae species and advanced eusocial bumble bee species [55]) and ground-nesting species in the two habitats, with a binomial error distribution. Explanatory variables were the plant species (“*Lotus corniculatus*” vs. “*Sinapis alba*”), the habitat (“Urban” vs. “Rural”), the Julian day of the year (scaled) and their interactions. The sampling site and the year were included as random effects. Honey bees were excluded from these analyses.

Body size of wild bees was measured using a Zeiss stereomicroscope (Zeiss Discovery V12) with an integrated camera (AxioCam ICc5) to record pictures of each pinned specimen, using the software ZEN 2012 (blue edition) (all distributed by Carl Zeiss Microscopy GmbH, Jena, Germany). Inter-tegular distance (hereafter ITD), which is the standard measurement of body size in bees, was measured with the software imageJ version 1.52a [56]. Analyses of ITD variations over time in the two habitats were carried out separately for bumble bees and other wild bee taxa. Indeed, bumble bees are eusocial species, with important body size differences among different categories of individuals (queens, drones, workers) [57], and whose abundances vary along the course of the active season. In addition, they have an endogenous thermoregulation capacity, which can play a role in the temperature–height relationship [28,29,58]. On the contrary, all other wild bees sampled were either solitary species or more “primitively” eusocial species [55] with little to no social status-related variations in body size within the species. Log-transformed ITDs of the specimens were analyzed through glmms in a similar way to trait proportions, with a Gaussian error distribution. As a unimodal trend over time was expected for the ITD variations, we treated the Julian day as an orthogonal degree-2 polynome.

**Figure A2:**
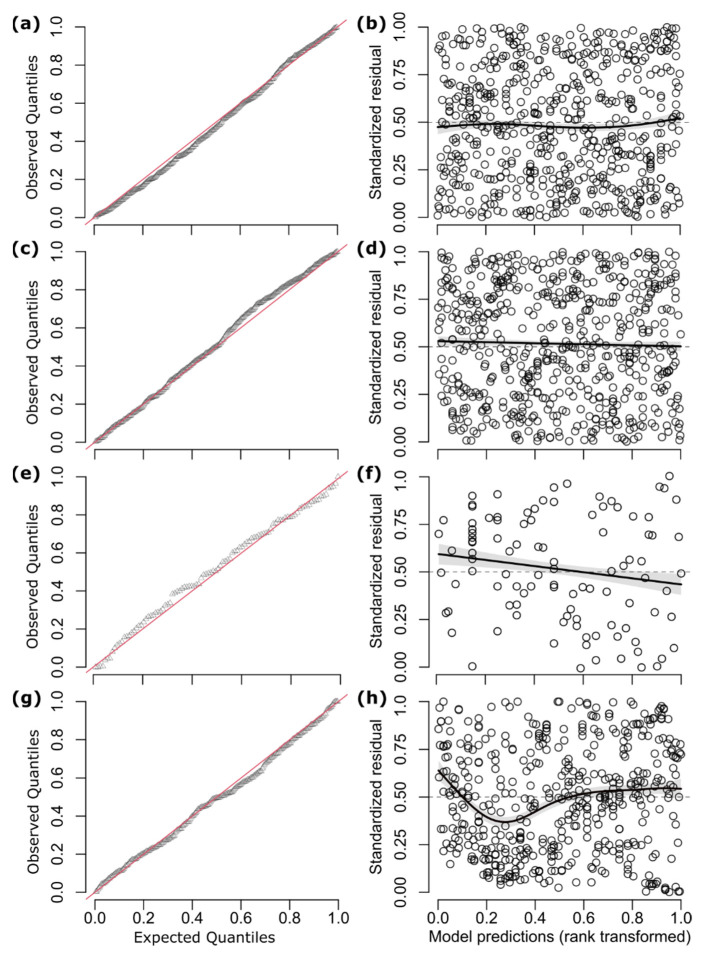
Residual diagnostic plots for glmms of trait variations over the year in both habitats and on both plants, as presented in Figure 2A: (**a**,**b**) glmm of the proportions of individuals belonging to eusocial species; (**c**,**d**) glmm of the proportion of individuals belonging to ground-nesting species; (**e**,**f**) glmm of ITD (mm) of *Bombus* specimens; (**g**,**h**) glmm of ITD (mm) of other wild bee specimens. The qq-plots (left panels) detect no overall deviations from the expected residual distribution, while the residuals vs. predicted values plots (right panels) detect no clear deviation from uniformity (the black curve represents the median of standardized residuals estimated by quantile regression).

Residuals of all glmms were inspected with the “DHARMa” package [59] and no deviations from the specified error distributions were detected (Figure A2). The contribution of interactions to models was tested through type-III tests with the “Anova” function (“car” package [60]). Model selection was performed on the basis of the second-order Akaike information criterion (AICc, “MuMIn” package [61]), which is used instead of the AIC when the sample size is small in comparison to the number of estimated parameters. Absence of collinearity was verified via the “check_collinearity” function (“performance” package [62]).

## 3. Results

### 3.1. Pollinator Diversity

In total, 888 individuals were captured, with fewer flower visitors in the rural habitat (360 specimens belonging to 74 species, among which 86% of individuals on *S. alba* and 14% on *L. corniculatus*) than in the urban habitat (528 specimens belonging to 63 species, among which 78% of individuals on *S. alba* and 22% on *L. corniculatus*) (Figure 1). However, flower visitor assemblages in the urban habitat were dominated by a small number of species with large populations. Thus, only six species of pollinators (in descending importance, the bee species *Bombus pascuorum*, *Apis mellifera*, *Andrena pusilla*, *Lasioglossum morio*; and the syrphid fly species *Syritta pipiens*, *Eupeodes luniger*) contributed to more than half of the specimens collected on the two plants in the urban habitat (Figure 1, Table 1).

As expected, flower visitor assemblages differed between the two plant species and between habitats. Hence, the main families of pollinators were represented in different proportions between the two habitats. On *S. alba*, Andrenidae (urban: 23%, rural: 8%) and Apidae (urban: 19%, rural: 5%) were relatively more abundant in the urban habitat than in the rural one, while it was the opposite for Colletidae (urban: 3%, rural: 11%), Halictidae (urban: 26%, rural: 43%) and Syrphidae (urban: 26%, rural: 30%) (χ²_df = 4_ = 82.98, *p* < 2.2 × 10^−16^) (Figure 1). Similarly, on *L. corniculatus*, Apidae (urban: 56%, rural: 26%) were dominant in the urban habitat, whereas Halictidae (urban: 6%, rural: 18%) and Megachilidae (urban: 28%, rural: 42%) were proportionally more numerous in the rural habitat (χ²_df = 2_ = 14.34, *p* = 0.00076). In the urban habitat, the most abundant species visiting *S. alba* were *Apis mellifera* (with 14% of all visits) and *Andrena pusilla* (12%), while in the rural habitat, *Lasioglossum politum* (9%) and *Lasioglossum aeratum* (9%) were more numerous. On *L. corniculatus*, visits were largely dominated by *Bombus pascuorum* in both habitats, though in distinct proportions (53% in urban and 18% in rural), followed by solitary bees of the *Megachile* genus (*M. willuhgbiella* in urban, 10%; *M. centuncularis* in rural, 12%) (Table 1).

Species richness of insect visitors of *S. alba* over the two years was higher in the rural habitat (N_rural_ = 67) than in the urban habitat (N_urban_ = 53) (Figure A1). In contrast, the urban habitat displayed overall more species visiting *L. corniculatus* (N_urban_ = 24) than the rural habitat (N_rural_ = 18). For both plant species, glmms returned no significant difference in species richness between urban and rural sites (*S. alba*: mean richness per site and year ± SE: n_rural_ = 22.3 ± 2.0 vs. n_urban_ = 25.5 ± 2.2, df = 15, t = −1.15, *p* = 0.27; *L. corniculatus*: n_rural_ = 6.3 ± 0.9 vs. n_urban_ = 7.2 ± 1.0; df = 15; t = −1.15; *p* = 0.27; Table A2). Regarding specific diversity, Simpson’s diversity index λ of *S. alba* visitors did not differ significantly between the two habitats (mean Simpson index per site and year ± SE: λ_rural_ = 0.91 ± 0.03 vs. λ_urban_ = 0.89 ± 0.03; df = 13; t = 0.40; *p* = 0.69; Table A2). However, Simpson’s diversity index of *L. corniculatus* visitors was significantly higher in the rural habitat (λ_rural_ = 0.75 ± 0.03 vs. λ_urban_ = 0.64 ± 0.03; df = 13; t = 2.73; *p* = 0.017, Table A2), suggesting greater evenness in the abundance distribution of species visiting this plant in the rural habitat.

There were only two specimens in the rural habitat with an “NT” (near threatened) IUCN conservation status [52]: they belonged to the species *Lasioglossum laevigatum* and *Lasioglossum monstrificum* and were foraging on *S. alba*. Moreover, we found six species whose conservation status is of importance in the Ile-de-France region (five bee species and one syrphid fly species) [53,54], meaning their presence is evidence for a noteworthy natural habitat (ZNIEFF). These species were more abundant in the rural habitat (five out of the six species mentioned above, representing 31 out of 32 specimens), and 84% of them were foraging on *S. alba*.

### 3.2. Wild Bees’ Functional Traits

As for the functional diversity of wild bees, there was no significant difference between rural and urban habitats, either on *S. alba* (mean Rao’s quadratic entropy per site and year ± SE: Q_rural_ = 0.033 ± 0.007 vs. Q_urban_ = 0.033 ± 0.007; df = 13; t = 0.06; *p* = 0.95) or on *L. corniculatus* (Q_rural_ = 0.39 ± 0.007 vs. Q_urban_ = 0.22 ± 0.007; df = 13; t = 1.73; *p* = 0.11) (Table A2).

There was no statistically detectable difference in the degree of lectism between the two habitats, with almost exclusively individuals belonging to polylectic species in both habitats. Only two species of oligolectic bees were observed, exclusively on *S. alba*: *Andrena lagopus* (four individuals in the urban habitat and one in the rural habitat), and *Chelostoma campanularum* (only one individual in the urban habitat). As for brood parasites, they were only found in the rural habitat on *S. alba* (1.9% of individuals on this plant in this habitat) and belonged to the genera *Nomada* (Apidae) and *Sphecodes* (Halictidae).

**Figure 2:**
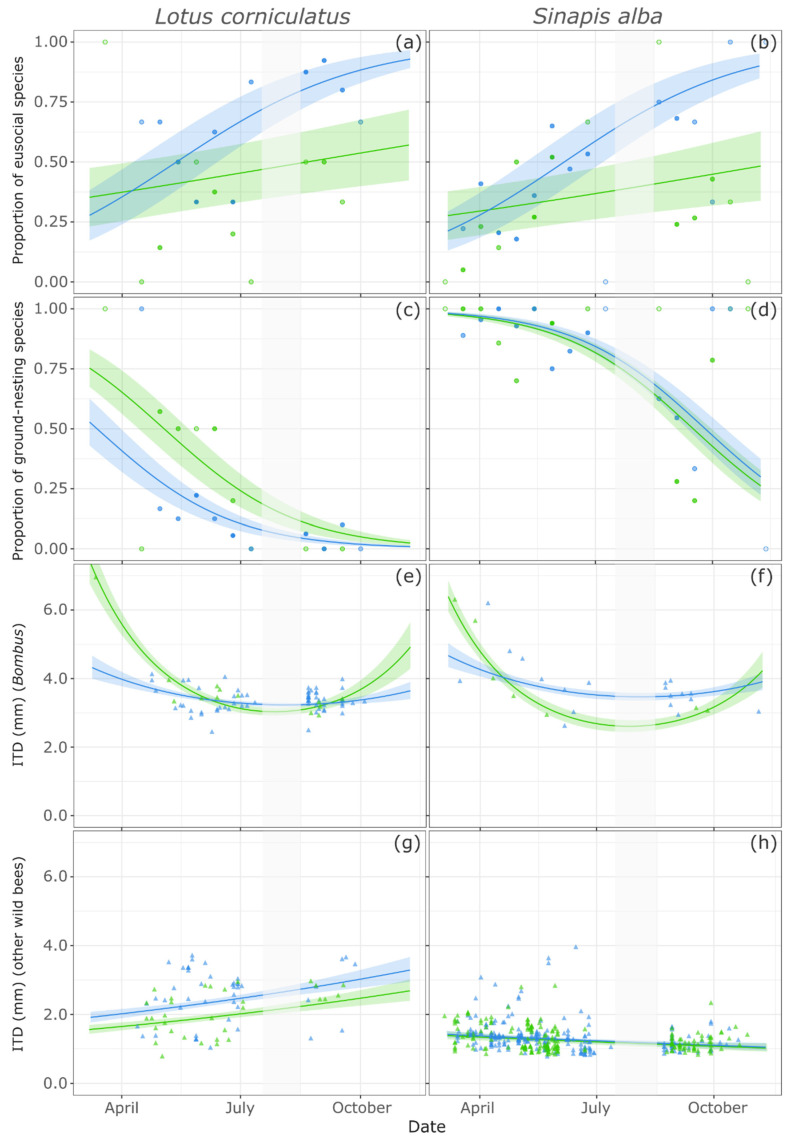
Trait variations throughout the year in both habitats (Urban: blue; Rural: green), for insect pollinators visiting *L. corniculatus* (left panels) and *S. alba* (right panels): (**a**,**b**) proportion of individuals belonging to eusocial species; (**c**,**d**) proportion of individuals belonging to ground-nesting species; (**e**,**f**) inter-tegular distance (ITD, in mm) of *Bombus* specimens; (**g**,**h**) ITD (mm) of other wild bee specimens. Curves display predictions from the models (±SE), dots represent fortnightly mean proportion of traits (transparence level is proportional to the number of specimens collected over the period) and triangles show raw ITD values. The gray-shaded part of each figure corresponds to the period when sampling could not be carried out due to a severe heat wave.

Sociality levels varied between the two habitats, and over the course of the season: the proportion of eusocial species (“primitive” or “advanced” sociality [55]) increased in the urban habitat on the two plants species (Figure 2a,b; Table 2). In contrast, this proportion remained stable in the rural habitat, at a low level and not significantly different from zero for both plant species (on *S. alba*: intercept estimate = −0.61 ± 0.47, *p* = 0.18; on *L. corniculatus*: intercept estimate = −0.25 ± 0.49, *p* = 0.61).

Concerning nesting habit variations over time, the proportion of ground-nesting species decreased in both habitats on the two plants species (Figure 2c,d; Table 2). On *L. corniculatus*, this proportion remained significantly higher in the rural habitat than in the urban one throughout the season (estimated intercept difference between habitats = 1.00 ± 0.50, *p* = 0.043). In contrast, there was no difference in the proportion of ground-nesting species visiting *S. alba* between the two habitats (estimated intercept difference between habitats = 0.18 ± 0.34, *p* = 0.59).

On both plant species, in the rural habitat, the ITD of bumble bees followed a unimodal seasonal pattern with a decrease during summer (Figure 2e,f; Table 2). However, this seasonal trend was less pronounced in the urban habitat on both plants. Additionally, bumble bees foraging on *S. alba* in the urban habitat had overall larger ITDs than those in the rural habitat (estimated intercept difference between habitats = 0.19 ± 0.06, *p* = 0.0013). As for other wild bees (solitary or “primitively” eusocial), no such unimodal seasonal pattern was detected. Indeed, the ITD of specimens visiting *L. corniculatus* steadily increased over time and was consistently and significantly higher in the urban habitat (Figure 2g; Table 2). On the contrary, the ITD of specimens visiting *S. alba* decreased over time, with no difference between habitats (Figure 2h; Table 2).

## 4. Discussion

As hypothesized, the pollinator assemblages visiting each plant species were different between the urban and the rural habitat. As expected, there were overall more species visiting the plant patches in the rural habitat, though species richness did not significantly differ between urban and rural sites. Further, as expected, the urban pollinator assemblage was dominated by two eusocial species: the managed honey bee (*Apis mellifera*) and one species of bumble bee (*Bombus pascuorum*), that mainly visited *S. alba* and *L. corniculatus*, respectively. Altogether, all species encountered in the urban habitat were very common species, not benefiting from any conservation status.

Moreover, in accordance with our second set of hypotheses, we recorded differences in functional diversity levels between the two habitats. Consistent with expectations, more eusocial, polylectic or above-ground nesting species were found in the urban habitat than in the rural one. Further, as expected, parasitic species were found solely in the rural habitat. However, these contrasts in functional diversity did not clearly translate into significant differences in the functional diversity estimator, Rao’s quadratic entropy, between the two habitats. Concerning body size variations, a seasonal unimodal trend (with smaller bees during the warmest months) was recorded for bumble bees in both habitats, although less pronounced in the urban habitat, thus contradicting our hypothesis of stronger constraints on body size in the urban habitat because of the urban heat island. Last, the body size of other wild bees foraging on *L. corniculatus* was larger in the urban habitat. There was no unimodal seasonal pattern of body size, but contrasting trends were recorded between the two plant species: body size of wild bees visiting the specialist *L. corniculatus* increased with time, whereas it slightly decreased for *S. alba.*

Overall, our rural habitat displayed either richer or more diverse assemblages than our very dense urban habitat, with more species representing more taxa, and among which some species have special conservation status. This was expected and in agreement with other studies comparing rural and dense urban habitats [4,5,7,8]. In particular, the scarcity of some taxa in the urban habitat, such as the Coleoptera and Lepidoptera orders, is a reminder that although urbanization has a general negative effect on the diversity of arthropods, not all taxa respond in the same way [2].

Nevertheless, we found more numerous flower visitors in the urban habitat than in the rural one. This trend could be partially explained by the dominance of eusocial species inducing high local abundance, a result already observed in other European cities [33]. In our results, specific and functional diversity patterns of the urban habitat were substantially affected by the over-representation of honey bees and bumble bees (mainly *B. pascuorum*). This translated into lower values of Simpson’s diversity index for this habitat. In particular, the high abundance of honey bees in urban areas can be explained by the intense beekeeping activity, with hive densities in Paris (26.14 hives/ km²) far exceeding those of the Seine-et-Marne department (1.90 hives/ km²) [63]. In fact, there is local experimental evidence that elevated hive densities are associated with lower visit frequencies of several wild pollinator categories [64].

In the Ile-de-France biogeographic region, Halictidae and Megachilidae have previously been described as urbanophilic families, whereas Syrphidae, Apidae and Colletidae have been designated as urbanoneutral and Andrenidae as urbanophobic [9]. Here, we indeed detected a higher proportion of Megachilidae in the urban than in the rural habitat, but many families presented different patterns: Andrenidae and Apidae were also well represented in urban habitats, the second one due to eusocial species. In addition, Syrphidae and Colletidae individuals were proportionally more abundant in the rural habitat. Overall, this suggests that the family level may not be relevant to accurately assess the tolerance of pollinators to urbanization.

In the urban habitat, we observed proportionally more eusocial individuals and fewer ground-nesting individuals than in the urban one. This is consistent with our predictions as well as other studies. Eusocial species may be advantaged in the city as they better cope with times of scarcity [55,65], while ground-nesters may struggle to find suitable nesting places in impervious urban landscapes [16,66,67]. On the contrary, cavity-nesting bee species may benefit from a wide variety of suitable nesting opportunities in the urban habitat, thus granting them a better tolerance to urbanization [8,9,15,68,69]. As for the temporal patterns of these traits, they differed little between the two habitats. Moreover, the proportion of individuals of eusocial species increased through the season. This could be explained by an increase in bumble bee abundance during late spring and summer, as the colonies produce more and more workers [70,71]. The same could be true for primitively eusocial Halictidae species, although worker numbers are often lower [55,72]. On the contrary, ground-nesting bees were more numerous at the start of the season, as they were mainly represented by the genus *Andrena*, which comprises early-emerging bees [73].

There was no clear difference in the degree of lectism between habitats or plants, nor any detectable temporal trend. We observed a vast majority of polylectic individuals similarly in both habitats. This result cannot be generalized since it can be explained by the low diversity of the experimental plant assemblage. As it comprised only two species, it attracted only a portion of the bee community. Likewise, brood parasitic species were scarce, and all of them were found in the rural habitat, in agreement with other studies [74,75]. This is consistent with the use of brood parasitic bees as a good indicator of the diversity of bee assemblages, as each parasitic species is dependent on finding its specific host. However, our data are too limited to suggest the absence of brood parasites in cities. Brood parasitic species have been documented in urban areas by studies with larger samples [8,66]. These results only support the hypothesis that such species are more abundant in rural habitats.

Our two plant species proved to be complementary in the study of pollinator communities, since the assemblages visiting them were quite different. This supports their use as a limited proxy for comparisons of pollinator communities across habitats. *Sinapis alba* (Brassicaceae) is a generalist species with floral resources easily accessible to a wide range of flower visitors (Figure 1). Noteworthy, this plant attracted oligolectic bees, specialized in foraging not only on Brassicaceae (*Andrena lagopus*), but also, curiously, on Campanulaceae (*Chelostoma campanularum*). It also attracted species with special conservation status, but mostly in the rural habitat. In contrast, *Lotus corniculatus* (Fabaceae) has a narrower set of flower visitors because its floral resources are only accessible to insects with long mouthparts and a body size large enough to access the floral rewards (Figure 1). There were more species visiting this plant in the urban habitat, as long-tongued bee species might be more tolerant to urbanization [8,9]. Several bumble bee species are reported to flourish in the city [33,65]; hence, more than 50% of all individuals foraging on *L. corniculatus* belonged to a single *Bombus* species. As for the other genera visiting this plant, they exhibited greater ITD values than those of *S. alba* visitors, since long mouthparts are associated with large body size (Figure 2g,h).

Overall, the ITDs of bumble bees visiting *S. alba* and other wild bees visiting *L. corniculatus* were larger in the urban habitat. This result refutes our hypothesis, as we expected smaller body size as a response to limited resources and higher temperatures in the city [14,15,24]. Instead, it is consistent with recent evidence of an increase in bumble bee size with urbanization at the species and community levels [76]. Would a large size be advantageous because it enables greater flying distances in a fragmented environment [22]? As a matter of fact, a study of wild bees in a U.S. city [77] showed that large bees benefit more than small bees from high floral density in an impervious and hot urban habitat. Hence, large bees may be more tolerant to fragmented urban landscapes, provided that they find locally abundant resources in green spaces. Furthermore, the body size of bumble bee queens increased over the last century in Belgium, linked to the increasing fragmentation and urbanization of the habitats [58]. This occurred despite the general rise in temperature, as bumble bees follow a size–temperature relationship opposite to Bergmann’s rule, which is most likely the result of their partial endothermy [28]. The effect of putative diminished resources and the heat island on wild bee body size in the city appears, therefore, less pronounced than expected. It could be mitigated by opposite selective pressures related to habitat fragmentation.

In previous studies, wild bee body size seemed nonetheless affected by seasonal changes in accordance with Bergmann’s rule, with a community-level size decrease during the warmer months [29]. While we expected a seasonal decrease in wild bee body sizes and ITDs, we found this trend only for bumble bees, with a more pronounced summer decrease in rural areas. In early colonies, bumble bee worker larvae are often poorly fed and thus develop into smaller adults [78,79]. A period of floral resource shortage may also explain the summer decrease in size at the community level. Such seasonal deficits in nectar supply to bumble bees have been demonstrated in English farmlands [80]. Meanwhile, in urban habitats, seasonal resource variations might be buffered by ornamental flora [31] and watering of flower beds, hence the less pronounced seasonal decrease in body size we observed in urban sites. In addition, when measuring the ITDs of bumble bees, we did not discriminate between bumble bee castes. Therefore, in our urban habitat, these buffered seasonal size variations might also be linked to altered phenology patterns of bumble bee colonies. Indeed, year-round activity of bumble bee queens and workers has been recorded in some urban habitats, suggesting more generations within a year [30]. Thus, in urban sites, overlapping generations and the extended presence of larger-bodied reproductive castes—queens and males—may have attenuated the body size seasonal trend. On the contrary, in the rural habitat, large individuals belonging to reproductive castes emerge at the beginning and end of the season and may be responsible for strong seasonal variations in the ITD. Such results should be interpreted with caution, though, given the low overall number of bumble bees captured in the rural habitat.

In contrast, the ITD of other wild bees visiting *L. corniculatus* and *S. alba* changed linearly over time in both habitats, increasing and decreasing, respectively. To our knowledge, these linear trends over the course of a season have not been recorded yet. In our data, we thus observed an increasing difference between the mean size of bees visiting the two plant species throughout our sampling period. One hypothesis to explain this trend would be that open flowers with easily accessible floral rewards are becoming increasingly available towards the end of summer (peak flowering of some Apiaceae and Asteraceae such as *Daucus carota*, *Helminthotheca echioides*, *Picris hieracioides* [42]). Small bees (e.g., the genera *Hylaeus* and *Lasioglossum*), which also visit *S. alba*, could benefit from this resource and consequently be more abundant during this period. Meanwhile, apart from bumble bees (Apidae), *L. corniculatus* was mainly visited by large Megachilidae bees (e.g., the genera *Megachile*, *Anthidium* and *Anthidiellum*) (Figure 1) that appeared late in the season.

## 5. Conclusions

There was a strong urban signature on the taxonomic and functional diversity of pollinator assemblages visiting our experimental plant setup. Our sampling method offers an alternative to colored pan traps, allowing accurately and exclusively collecting flower visitors in a standardized way across habitats and seasons. Here, the manipulated flowering phenology of the plants allowed detecting significant within-year changes in functional diversity. However, this method requires more time as well as more financial and human resources and limits sampling to visitors of a restricted array of plant species. In the city, we found lower taxonomic diversity, lower seasonal variation in bumble bee body size and lower abundance of brood parasitic and ground-nesting bees, but overall more individuals and larger bees. This suggests that our dense urban environment can be suitable for some bees, but only a few bee species really benefit from urban functional filtering. Trends in body size in the city could be a response to habitat fragmentation combined with a weakening of seasonal constraints, though it remains to be investigated in future research. Such consideration for seasonality and connectivity between resources embedded in urban landscapes is growing in the literature [15,31,81], as it appears to be relevant in understanding how to preserve pollinator functional diversity in the city. This could be a key factor in maintaining the pollination function despite the impacts of global changes—including climate change and increasing urbanization of habitats—on insect pollinator communities.

## Figures and Tables

**Table 1:** List of species and corresponding numbers of captured individuals in the two habitats (Rur.: Rural, Urb.: Urban).

List of Species	Rur.	Urb.	List of Species (Continued)	Rur.	Urb.
**HYMENOPTERA (BEES)**			**HYMENOPTERA (OTHERS)**		
**Andrenidae**			**Crabronidae**		
*Andrena apicata* (Smith 1847)	1		*Cerceris sp.*		1
*Andrena bicolor* (Fabricius 1775)	2	5	*Pemphredon sp.*	1	
*Andrena bimaculata* (Kirby 1802)	2		**Vespidae**		
*Andrena cineraria* (Linnaeus 1758)		2	*Vespula germanica* (Fabricius 1793)		1
*Andrena dorsata* (Kirby 1802)	4	3	**Tenthredinidae**		
*Andrena flavipes* (Panzer 1799)	4	2	*Allantus sp.*	1	
*Andrena gravida* (Imhoff 1832)		4	*Athalia sp.*	1	
*Andrena haemorrhoa* (Fabricius 1781)		1	*Cladius sp.*		1
*Andrena lagopus* (Latreille 1809)	1	4	*Tenthredo sp.*	1	
*Andrena minutula* (Kirby 1802)	5	12	**COLEOPTERA**		
*Andrena minutuloides* (Perkins 1914)		3	**Chrysomelidae**		
*Andrena pusilla* (Pérez 1903)	4	47	*Cryptocephalus sp.*	1	
*Andrena sp.*		1	*Psylliodes sp.*	1	
*Andrena subopaca* (Nylander 1848)	2	1	**Oedemeridae**		
*Andrena tenuistriata* (Pérez 1895)		14	*Oedemera nobilis* (Scopoli 1763)	1	
**Apidae**			*Oedemera sp.*	2	
*Anthophora quadrimaculata* (Panzer 1789)		1	**Scarabaeidae**		
*Apis mellifera* (Linnaeus 1758)	2	59	*Phyllopertha horticola* (Linnaeus 1758)	2	
*Bombus lapidarius* (Linnaeus 1758)	4		**DIPTERA**		
*Bombus lucorum* (Linnaeus 1760)	1	2	**Bombyliidae**		
*Bombus pascuorum* (Scopoli 1763)	14	77	*Bombylius major* (Linnaeus 1758)	3	2
*Bombus terrestris* (Linnaeus 1758)	1	3	**Conopidae**		
*Nomada bifasciata* (Olivier 1811)	1		*Myopa sp*	1	
*Nomada ferruginata* (Linnaeus 1767)	1		**Muscidae**		
*Nomada flavoguttata* (Kirby 1802)	1		*Phaonia sp.*		2
*Nomada fulvicornis* (Fabricius 1793)	1		**Scathophagidae**		
*Nomada panzeri* (Lepeletier 1841)	1		*Cordilura sp.*		1
**Colletidae**			*Scathophaga sp.*	1	
*Hylaeus brevicornis* (Nylander 1852)	6		**Syrphidae**		
*Hylaeus communis* (Nylander 1852)	16	3	*Brachypalpus valgus* (Panzer 1798)	1	
*Hylaeus gibbus* (Saunders 1850)	1		*Cheilosia fasciata* (Schiner & Egger 1853)	1	
*Hylaeus pictipes* (Nylander 1852)		5	*Cheilosia sp.*	1	
*Hylaeus punctatus* (Brullé 1832)	9	7	*Episyrphus balteatus* (De Geer 1776)	21	22
**Halictidae**			*Eristalis arbustorum* (Linnaeus 1758)	3	1
*Halictus scabiosae* (Rossi 1790)	1		*Eristalis tenax* (Linnaeus 1758)	10	10
*Halictus subauratus* (Rossi 1792)	5	4	*Eupeodes corollae* (Fabricius 1794)	6	1
*Halictus submediterranea* (Pauly 2015)	1		*Eupeodes luniger* (Meigen 1822)		25
*Halictus tumulorum* (Linnaeus 1758)	5	9	*Helophilus pendulus* (Linnaeus 1758)	1	
*Lasioglossum aeratum* (Kirby 1802)	28		*Melanostoma mellinum* (Linnaeus 1758)	3	3
*Lasioglossum albipes* (Fabricius 1781)	14		*Melanostoma scalare* (Fabricius 1794)		2
*Lasioglossum calceatum* (Scopoli 1763)	12	9	*Meliscaeva auricollis* (Meigen 1822)	4	
*Lasioglossum glabriusculum* (Morawitz 1872)		1	*Merodon equestris* (Fabricius 1794)	1	1
*Lasioglossum laevigatum* (Kirby 1802)	1		*Myathropa florea* (Linnaeus 1758)		1
*Lasioglossum laticeps* (Schenk 1868)	4	18	*Neoascia podagrica* (Fabricius 1775)		1
*Lasioglossum leucozonium* (Schrank 1781)	2		*Paragus haemorrhus* (Meigen 1822)	1	
*Lasioglossum limbellum* (Morawitz 1876)		1	*Paragus quadrifasciatus* (Meigen 1822)		1
*Lasioglossum malachurum* (Kirby 1802)		7	*Parasyrphus punctulatus* (Verrall 1873)	1	
*Lasioglossum marginatum* (Brullé 1832)	2	1	*Platycheirus albimanus* (Fabricius 1781)	1	1
*Lasioglossum minutissimum* (Kirby 1802)	3	5	*Platycheirus scutatus* (Meigen 1822)		4
*Lasioglossum monstrificum* (Morawitz 1891)	1		*Scaeva pyrastri* (Linnaeus 1758)	1	
*Lasioglossum morio* (Fabricius 1793)	17	39	*Sphaerophoria scripta* (Linnaeus 1758)	10	5
*Lasioglossum nitidiusculum* (Kirby 1802)		1	*Syritta pipiens* (Linnaeus 1758)	7	34
*Lasioglossum nitidulum* (Fabricius 1804)	3	7	*Syrphus sp.*	1	
*Lasioglossum pallens* (Brullé 1832)	6		*Syrphus nitidifrons* (Becker 1921)	2	
*Lasioglossum pauxillum* (Schenck 1853)		2	*Syrphus torvus* (Osten-Sacken 1875)	17	
*Lasioglossum politum* (Schenck 1853)	27	7	**Tachinidae**		
*Lasioglossum punctatissimum* (Schenck 1853)	5		*Tachina sp.*		1
*Lasioglossum villosulum* (Kirby 1802)		1	**LEPIDOPTERA**		
*Sphecodes puncticeps* (Thomson 1870)	1		**Lycaenidae**		
**Megachilidae**			*Aricia agestis* (Denis & Schiffermüller 1775)	2	
*Anthidiellum strigatum* (Panzer 1805)	3	2	**Pieridae**		
*Anthidium oblongatum* (Illiger 1806)		1	*Gonepteryx rhamni* (Linnaeus 1758)	1	
*Anthidium punctatum* (Latreille 1809)	4		*Pieris rapae* (Linnaeus 1758)	1	2
*Chelostoma campanularum* (Kirby 1802)		1			
*Hoplitis leucomelana* (Kirby 1802)	1	1			
*Megachile centuncularis* (Linnaeus 1758)	6	11			
*Megachile willughbiella* (Kirby 1802)		15			
*Osmia bicornis* (Linnaeus 1758)	1	1			
*Osmia caerulescens* (Linnaeus 1758)	4	4			
*Osmia cornuta* (Latreille 1805)		1			
*Osmia submicans* (Morawitz 1870)	5				

**Table 2:** Equations of the models of trait variations over time in both habitats. Equations are composed of the estimated parameters with the standard errors in brackets. NS means that the estimator is not significantly different from 0; parameters in bold indicate a significant difference between the two habitats (significance level: 5%). DAY: Julian day of the year.

Modeled Trait Variable	Plant	Habitat	Equation
Proportion of eusocial species (GLMM, Binomial family)	*L. corniculatus*	Rural	−0.25 (0.49, NS) + 5.22 (3.36, NS) × DAY
		Urban	0.45 (0.47, NS) + **20.61** (3.32) × DAY
	*S. alba*	Rural	−0.61 (0.47, NS) + 5.22 (3.36, NS) × DAY
		Urban	0.09 (0.45, NS) + **20.61** (3.32) × DAY
Proportion of ground-nesting species (GLMM, Binomial family)	*L. corniculatus*	Rural	**−0.80** (0.37) + −28.02 (3.20) × DAY
		Urban	**−1.80** (0.33) + −28.02 (3.20) × DAY
	*S. alba*	Rural	1.85 (0.26) + −28.02 (3.20) × DAY
		Urban	2.03 (0.25) + −28.02 (3.20) × DAY
ITD (log) genus *Bombus* (LMM, Gaussian family)	*L. corniculatus*	Rural	1.25 (0.03) + **−1.12** (0.24) × DAY + **1.31** (0.24) × DAY²
		Urban	1.22 (0.01) + **−0.46** (0.14) × DAY + **0.39** (0.15) × DAY²
	*S. alba*	Rural	**1.10** (0.05) + **−1.12** (0.24) × DAY + **1.31** (0.24) × DAY²
		Urban	**1.29** (0.03) + **−0.46** (0.14) × DAY + **0.39** (0.15) × DAY²
ITD (log) Other wild bees (LMM, Gaussian family)	*L. corniculatus*	Rural	**0.65** (0.05) + 2.81 (0.89) × DAY
		Urban	**0.85** (0.04) + 2.81 (0.89) × DAY
	*S. alba*	Rural	0.21 (0.02) + −1.56 (0.29) × DAY
		Urban	0.24 (0.02) + −1.56 (0.29) × DAY

## Data Availability

All data are archived in the publicly accessible repository Zenodo, within the “iEES-Paris OpenData” community: https://doi.org/10.5281/zenodo.4564318 accessed on 26 February 2021.

## Appendix A

**Table A1:** Proportions of land use categories [38] within a radius of 500 m around the experimental sites (SEF: Station d’Ecologie Forestière of Fontainebleau-Avon; CEREEP A and B: CEREEP-Ecotron Ile-de-France; SU: Pierre et Marie Curie campus of Sorbonne Université; JDP: Jardin des Plantes; CIUP: Cité Internationale Universitaire of Paris).

Site	Coordinates	Continuous Urban Landscape (%)	Discontinuous Suburban Landscape (%)	Urban Green Spaces (%)	Permanent Grassland (%)	Forests (%)
**RURAL**						
CEREEP A (2017)	48.2867° N, 2.6781° E	8	26	0	2	64
CEREEP B (2017–2018)	48.2831° N, 2.6657° E	0	0	0	49	51
SEF (2017–2018)	48.4206° N, 2.7289° E	0	43	0	0	57
**URBAN**						
CIUP (2017)	48.8189° N, 2.3353° E	48	16	36	0	0
JDP (2017–2018)	48.8440° N, 2.3611° E	63	3	34	0	0
SU (2017–2018)	48.8465° N, 2.3587° E	68	0	32	0	0

**Table A2:** Mean comparison of diversity indices and Tukey post hoc test between the rural (Rur.) and urban (Urb.) habitats. Terms in bold indicate a significant difference between the two habitats (significance level: 5%).

Index	Plant	Mean ± SE (Across Sites and Years)		Estimator of Habitat Type		Tukey Post Hoc Test	
		Rural	Urban	Estimate ± SE	*p*	Direction	*p*
Species richness (GLMM, Poisson family)	*L. corniculatus*	6.29 ± 0.87	7.19 ± 0.97	0.13 ± 0.12	0.25		0.27
	*S. alba*	22.29 ± 2.00	25.47 ± 2.16	0.13 ± 0.12	0.25		0.27
Simpson’s index (LMM)	*L. corniculatus*	0.747 ± 0.029	0.636 ± 0.029	−0.112 ± 0.041	0.0064	**Rur. > Urb.**	**0.017**
	*S. alba*	0.910 ± 0.029	0.894 ± 0.029	−0.017 ± 0.041	0.69		0.69
Rao’s quadratic entropy (LMM)	*L. corniculatus*	0.0394 ± 0.0072	0.0218 ± 0.0072	−0.018 ± 0.010	0.084		0.11
	*S. alba*	0.0334 ± 0.0072	0.0328 ± 0.0072	−0.001 ± 0.010	0.95		0.95
